# Early supplemental parenteral nutrition for the achievement of nutritional goals in subarachnoid hemorrhage patients: An observational cohort study

**DOI:** 10.1371/journal.pone.0265729

**Published:** 2022-03-18

**Authors:** Mario Kofler, Ronny Beer, Stephanie Marinoni, Alois J. Schiefecker, Maxime Gaasch, Verena Rass, Anna Lindner, Bogdan A. Lanosi, Paul Rhomberg, Bettina Pfausler, Claudius Thomé, John F. Stover, Erich Schmutzhard, Raimund Helbok

**Affiliations:** 1 Neurological Intensive Care Unit, Department of Neurology, Medical University of Innsbruck, Innsbruck, Austria; 2 Medical Informatics, UMIT–University for Health Sciences, Hall, Austria; 3 Department of Neuroradiology, Medical University of Innsbruck, Innsbruck, Austria; 4 Department of Neurosurgery, Medical University of Innsbruck, Innsbruck, Austria; 5 Fresenius Kabi Germany, Bad Homburg vor der Höhe, Germany; University of Vienna: Universitat Wien, AUSTRIA

## Abstract

**Purpose:**

Enteral nutrition (EN) often fails to achieve nutritional goals in neurocritical care patients. We sought to investigate the safety and utility of supplemental parenteral nutrition (PN) in subarachnoid hemorrhage (SAH) patients.

**Materials and methods:**

Data of 70 consecutive patients with non-traumatic SAH admitted to the neurological intensive care unit of a tertiary referral center were prospectively collected and retrospectively analyzed. We targeted the provision of 20–25 kilocalories per kilogram bodyweight per day (kcal/kg/d) by enteral nutrition. Supplemental PN was given when this target could not be reached. Nutritional data were analyzed for up to 14 days of ICU stay. Hospital complications were tested for associations with impaired enteral feeding. The amounts of EN and PN were tested for associations with the level of protein delivery and functional outcome. Repeated measurements within subjects were handled utilizing generalized estimating equations.

**Results:**

Forty (27 women and 13 men) of 70 screened patients were eligible for the analysis. Median age was 61 (IQR 49–71) years, 8 patients (20%) died in the hospital. Thirty-six patients (90%) received PN for a median duration of 8 (IQR 4–12) days. The provision of 20 kcal/kg by EN on at least 1 day of ICU stay was only achieved in 24 patients (60%). Hydrocephalus (p = 0.020), pneumonia (p = 0.037) and sepsis (p = 0.013) were associated with impaired enteral feeding. Neither the amount nor the duration of PN administration was associated with an increased risk of severe complications or poor outcome. Supplemental PN was associated with significantly increased protein delivery (p<0.001). In patients with sepsis or pneumonia, there was an association between higher protein delivery and good functional outcome (p<0.001 and p = 0.031), but not in the overall cohort (p = 0.08).

**Conclusions:**

Enteral feeding was insufficient to achieve nutritional goals in subarachnoid hemorrhage patients. Supplemental PN was safe and associated with increased protein delivery. A higher protein supply was associated with good functional outcome in patients who developed sepsis or pneumonia.

## Introduction

Appropriate nutrition goals for neurocritical care patients, including non-traumatic subarachnoid hemorrhage (SAH) patients, are not well known. International guidelines for nutrition in critically ill adults recommend the early initiation of enteral nutrition (EN) and, in traumatic brain injury (TBI) patients, immune-modulating formulations [1, 2]. In contrast to surgical and medical populations, there is evidence that enhanced caloric supply during the early phase of critical illness is associated with a decrease in mortality [3, 4].

Regarding the route of administration of artificial nutrition, no difference in mortality was found between patients receiving EN or parenteral nutrition (PN) in mixed populations of ICU patients [5, 6]. Yet EN is usually preferred over PN, endeavoring to maintain gut integrity [7]. However, enteral feeding often fails to achieve the provision of nutritional requirements in mixed populations [8], and also in subarachnoid hemorrhage (SAH) patients [9].

The benefit of supplemental PN to compensate for a lack of provided calories and protein as well as the optimal timing for its initiation are unclear, as trials have yielded conflicting results. A large observational study found an association between the use of supplemental PN and increased mortality and length of intensive care unit stay [10]. An interventional trial reported a lower incidence of infectious complications in patients receiving early supplemental PN [11]. Late initiation of supplemental PN (after 7 days) was associated with a reduced length of ICU stay, less infectious complications and a shorter duration of mechanical ventilation compared to the early initiation (after 3 days) [12], however, the applicability of these findings in clinical routine and different patient populations has been questioned [13]. A recent meta-analysis concluded that, if enteral feeding fails to fulfill nutritional requirements in ICU patients, supplemental PN is associated with a reduction of nosocomial infections and lower ICU mortality [14].

Adequate protein supply in critically ill patients has gained increased attention, as reaching protein goals appears to be even more important than the provision of caloric requirements [15, 16]. Higher protein doses were associated with decreased mortality [16–18], more ventilator-free days [19], an improvement of parameters indicating multi-organ failure [20], and preserved muscle strength [21]. Furthermore, the replication and function of immune cells rely on sufficient protein availability [22].

Studies on nutrition in SAH patients indicate that EN does not provide an adequate amount of calories and protein and that this lack of sufficient nutrition may be associated with a higher rate of hospital infections and poor outcome [9, 23, 24]. In traumatic brain injury (TBI) patients, increased caloric supply was associated with a decrease in mortality and hospital complications [4, 25]. Furthermore, in TBI patients, the early initiation of nutrition was associated with good outcome, irrespective of the route of administration [26]. These findings may argue for supplemental PN in neurocritical care patients if nutrition goals cannot be achieved by EN.

Our primary hypothesis was that enteral nutrition would be insufficient to provide adequate amounts of energy and protein and that supplemental parenteral nutrition would safely promote the accomplishment of nutrition goals.

## Materials and methods

### Patient and data selection

This is a retrospective analysis of data prospectively recorded from 70 consecutive SAH patients admitted to the neurocritical care unit at the Medical University of Innsbruck, Austria, between November 2011 and February 2013. The ethics committee of the Medical University of Innsbruck approved the conduct of this study (AN3898 285/4.8, AM4091-292/4.6) and informed consent was obtained from all patients according to federal regulations. The study was performed in accordance with the ethical standards as laid down in the 1964 Declaration of Helsinki and its later amendments. Inclusion criteria were (1) the diagnosis of spontaneous SAH, (2) patient age over 18 years, (3) the administration of artificial nutrition and (4) a full 24-hour dataset available for the analyzed days. Analysis of nutrition data was performed up to 14 days after the hemorrhage or until ICU discharge, oral food intake, death, or the time point of withholding nutrition as part of palliation. The patient exclusion process is provided as S1 Fig. The day of admission (referred to as day 0) was excluded, because it does not provide a 24-hour dataset.

### Patient care

Disease severity was graded using the Hunt and Hess scale, the Acute Physiology and Chronic Health Evaluation II (APACHE II) score and the modified Fisher Scale on admission computed tomography (CT) scan [27–29]. Clinical care of SAH patients was based on current international guidelines [30–32], with the exception of nimodipine being administered intravenously in intubated patients. Aneurysms were treated early, by either surgical clipping or endovascular coiling. Intravenous fluids (crystalloids or colloids), vasopressors (norepinephrine or phenylephrine) and dobutamine were used for hemodynamic stabilization. Vasopressor doses were converted into norepinephrine equivalents. Continuous intravenous midazolam and sufentanil were routinely used to facilitate mechanical ventilation. All patients were followed by transcranial color-coded duplex ultrasound. Delayed cerebral ischemia (DCI) was defined as either a new focal neurological deficit, a decrease of at least 2 points on the Glasgow Coma Scale, or a new infarct on CT or MRI, not attributable to other causes [33]. Functional outcome was assessed 3 months after the bleeding using the modified Rankin Scale (mRS). Scores of 0–2 were defined as good and scores of 3–6 as poor functional outcome.

### Infectious complications

Patients were screened daily for the development of infectious complications and the date of occurrence was recorded. The diagnosis was prospectively confirmed according to CDC and sepsis criteria weekly by the treating intensivists and members of the study team [34, 35]. The onset of pneumonia was classified as early (until day 3) or late (after day 3) after admission [36].

### Nutrition protocol

Early enteral feeding (within 24 hours after aneurysm treatment) was intended. Only nasogastric tubes were used for the administration of EN in this study. Isocaloric EN was initiated with a flow rate of 20–25 ml/h, which was gradually increased to target 20–25 kilocalories per kilogram bodyweight per day (kcal/kg/d) on day 4. Flow rate was adapted to gastrointestinal tolerance and therefore not increased if gastric residual volume (GRV), which was measured every 6 hours, exceeded 250 ml. During the initial increase of EN, patients received a daily intravenous amino acid infusion. If 80% of calculated calories (16 kcal/kg/d) could not be administered by EN by day 4, supplemental PN (an all-in-one emulsion of glucose, amino acids and lipids was added. In case of severe gastrointestinal intolerance, an earlier start of PN could be initiated at the discretion of the treating physician. If the patient’s body mass index (BMI) exceeded 30, the ideal bodyweight was used for the calculation of energy requirements instead of actual bodyweight. All calories administered with non-nutritional infusions were also quantified and considered in the “reached caloric goal analyses” (mainly propofol, 1.1 kcal/ml, and glucose, 3.4 kcal/g).

In the presence of gastrointestinal intolerance there was a stepwise approach: domperidone (10 mg every 8 h) was used as routine prokinetic agent. If 1 measurement of GRV was greater than 250 ml, metoclopramide (10 mg every 8 hours) could be added. If no response to metoclopramide was observed after 48 hours, erythromycin (250 mg every 6 hours) could be administered for a maximum of 4 days.

For statistical analysis we defined 3 nutrition therapy goals: (1) reaching 16 kcal/kg/d by EN at least once until day 4 (according to our nutrition protocol, (2) reaching 20 kcal/kg/d by EN at least once until day 7; and (3) reaching 20 kcal/kg/d by EN at least once until day 14.

### Safety

The safety of PN was assessed by associating the duration of application of PN and the amount of administered PN with important neurological (DCI), medical (anemia, acute renal failure, necessity of percutaneous dilatational tracheotomy) and infectious complications as well as length of mechanical ventilation and length of ICU stay.

### Data management and statistical analysis

Patient characteristics, radiographic data, hospital complications and functional outcome were prospectively recorded in our institutional SAH database. Nutrition data and medication were collected using the patient data management system (PDMS, Centricity Critical Care 7.0 SP2, GE Healthcare Information Technologies, Dornstadt, Germany) and pooled over 1 day.

Continuous variables are reported as mean and standard error of mean (SEM) or median and interquartile range (IQR). Categorical variables are reported as count and proportions in each group.

Time-series data were analyzed using a generalized linear model, using a normal distribution and identity link function, which was extended by generalized estimating equations (GEEs) with an autoregressive process of the first order to handle repeated measurements (days) within subjects. Individuals with missing cases were included. The type of model chosen for the GEE was dependent on the outcome variable: linear models were used for continuous variables, binary-logistic models for binary categorical variables. Analyses without repeated measurements were performed using a linear or binary-logistic regression model. Analyses were performed with IBM-SPSS V22.0 (SPSS Inc., Chicago, IL, USA). The significance level was set at a *p*-value of less than 0.05. For significant results, the odds ratio (OR) and the 95% confidence interval (95% CI) are given.

## Results

541 ICU days of 40 SAH patients were analyzed. Patient characteristics, hospital complications and functional outcome are summarized in Table 1. Aneurysms were treated by surgical clipping (n = 23, 57.5%) or endovascular coiling (n = 15, 37.5%). In 2 patients (5%) no aneurysm was detected despite repeated angiogram. Eight patients (20%) died during the ICU stay.

**Table 1:** Patient characteristics, interventions, complications and outcome.

Patient characteristics	n (%) or median (IQR)
Hunt and Hess grade (admission), n (%)	
1	1 (2.5)
2	2 (5)
3	13 (32.5)
4	6 (15)
5	18 (45)
Modified Fisher score (admission CT), n (%)	
1	2 (5)
2	3 (7.5)
3	13 (32.5)
4	22 (55)
Age, median (IQR)	61 (49–71)
Gender (female), n (%)	27 (67.5)
BMI, median (IQR)	25 (23–28)
Diabetes, n (%)	2 (5)
Loss of consciousness, n (%)	20 (50)
APACHEII score (admission), median (IQR)	17 (13–20)
SAH sum score, median (IQR)	26 (18–28)
IVH sum score, median (IQR)	5 (0–8)
Aneurysm size above 10 mm, n (%)	11 (27.5)
Global cerebral edema, n (%)	18 (45)
SAH-related parenchymal hematoma, n (%)	15 (37.5)
**Interventions, complications, outcome**	
Hemicraniectomy, n (%)	12 (30)
Aneurysm rebleeding, n (%)	6 (15)
Pneumonia, n (%)	24 (60)
Sepsis, n (%)	6 (15)
Ventriculitis, n (%)	2 (5)
Urinary tract infection, n (%)	8 (20)
Anemia requiring transfusion, n (%)	18 (45)
Delayed cerebral ischemia, n (%)	12 (30)
Hydrocephalus requiring EVD, n (%)	30 (75)
Acute renal failure, n (%)	3 (7.5)
Length of mechanical ventilation (days), median (IQR)	11 (7–15)
Percutaneous dilatational tracheostomy, n (%)	13 (33)
Length of ICU stay (days), median (IQR)	33 (22–48)
Modified Rankin scale after 3 months, n (%)	
0	2 (5)
1	5 (12.5)
2	5 (12.5)
3	4 (10)
4	6 (15)
5	8 (20)
6	10 (25)

BMI = body mass index; APACHE II = acute physiology and chronic health evaluation II; SAH = subarachnoid hemorrhage; IVH = intraventricular hemorrhage; EVD = external ventricular drain.

### Gastrointestinal intolerance

Thirty-two patients (80%) had gastrointestinal intolerance (at least one GRV >250 ml). In these patients, the median GRV was 100 (IQR 50–250) ml. All patients (100%) received domperidone, 19 (47.5%) received metoclopramide and 4 (10%) erythromycin. No patient had a pre-existing gastric or enteral pathology.

### Nutrition therapy goals

**Fig 1:**
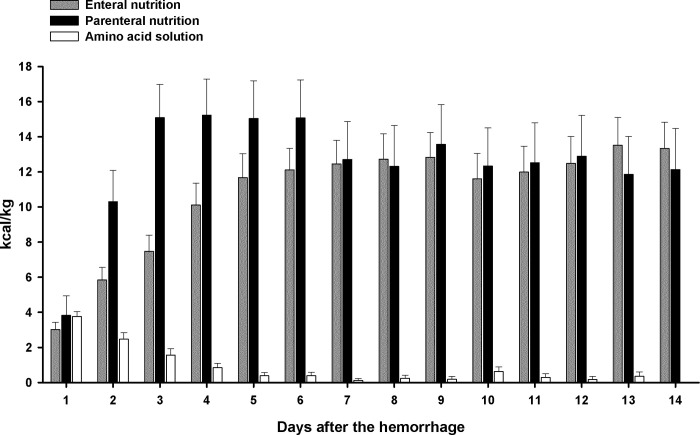
Fraction of provided calories by source. Daily mean kilocalories (kcal) per kilogram (kg) bodyweight provided by enteral nutrition, intravenous amino acid solutions (non-lipid-containing) and parenteral nutrition (lipid-containing) in 40 patients over 14 days following subarachnoid hemorrhage; error bars represent the standard error of mean.

Enteral feeding was started at a median of 1 day (IQR 1–2 days) after SAH. Thirty-six patients (90%) received supplemental PN, which was initiated at a median of 2 (IQR 1–3) days after the hemorrhage. In 18 patients (45%) the provision of 16 EN kcal/kg/d (80% of calculated requirements, goal 1) was achieved at least once by day 4; 17 patients (42.5%) had received 20 EN kcal/kg/d at least once by day 7 (goal 2); and in 24 patients (60%) the administration of 20 kcal/kg/d by EN was accomplished at least once until day 14 (goal 3). Due to the administration of supplemental PN, there was no difference in total administered calories whether or not the respective nutritional goals were reached: (1), p = 0.19; (2), p = 0.29; (3), p = 0.61. The sources of daily provided calories (EN, amino acid infusions and PN) are shown in Fig 1. By administering supplemental PN, the provision of 20 kcal/kg/d was quickly accomplished in all patients after a median of 2 (IQR 2–4) days.

### Factors associated with impaired enteral feeding

Hydrocephalus, pneumonia and sepsis were significantly associated with less EN (Table 2). Pneumonia was diagnosed in 24 patients (60%). Daily calories supplied by EN were not different between patients with early (n = 13, 54%) and late onset pneumonia (10.2±0.7 versus 9.4±0.6 kcal/kg/d, p = 0.85) and no difference in total administered calories (EN + PN) was found between patients with and without pneumonia (25.6±0.6 versus 26.1±0.6, p = 0.72). Higher doses of midazolam, sufentanil and vasopressors were also associated with less EN.

**Table 2:** Factors associated with impaired enteral feeding.

	Mean (SEM) of daily EN kcal				
Complication or intervention	*yes*	*no*	Adj. OR	95% CI	p-value
Hydrocephalus	9.6 ± 0.4	13.8 ± 0.8	0.82	0.71–0.97	0.020
Pneumonia	9.8 ± 0.5	11.9 ± 0.6	0.83	0.69–0.98	0.037
Sepsis	6.4 ± 0.8	11.3 ± 0.4	0.74	0.59–0.94	0.013
One versus no infectious complication	10.8 ± 0.5	13.8 ± 0.8	0.85	0.73–0.99	0.038
Two versus no infectious complications	7.1 ± 0.7	13.8 ± 0.8	0.67	0.54–0.82	<0.001
Sufentanil above median (178 mcg/h)	8.9 ± 0.5	12.4 ± 0.6	0.86	0.77–0.95	0.004
Midazolam above median (18 mg/h)	9.1 ± 0.4	12.8 ± 0.6	0.88	0.78–0.99	0.002
Norepinephrine equivalent dose (mcg/min)	n/a	n/a	0.99	0.99–1.00	0.046

Statistical analysis was performed using univariate linear models in generalized estimating equations with the amount of daily enteral nutrition (EN) kilocalories (kcal) as outcome variable. All analyses were adjusted for Hunt & Hess grade, modified Fisher grade, age, and gender. Additionally, the models including sufentanil, midazolam and norepinephrine equivalent dose were adjusted for the post-bleed day, as higher doses are given in the earlier stage of the disease, when EN is still being increased. n/a = not applicable.

### Parenteral nutrition and safety

**Fig 2:**
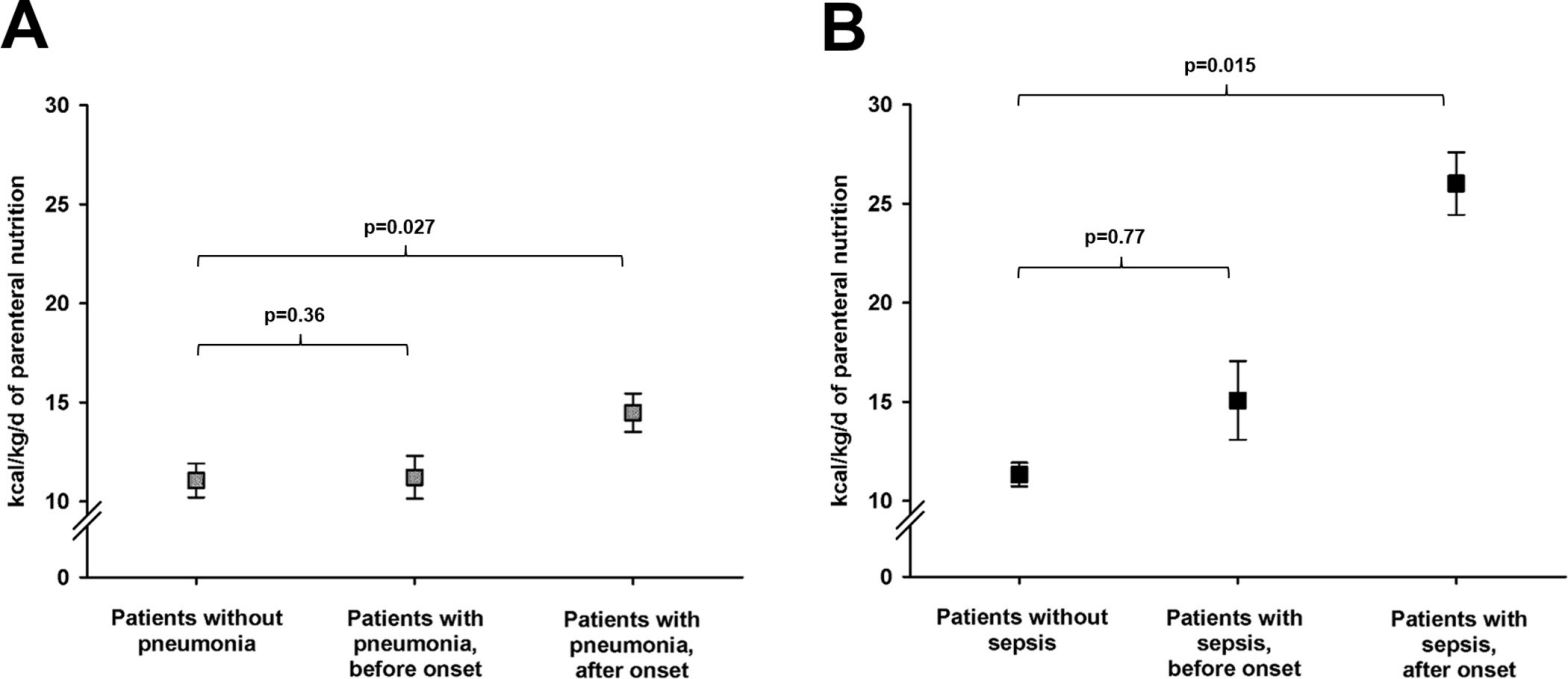
Amount of parenteral nutrition before and after the onset of severe infectious complications. Mean amount of supplemental parenteral nutrition (PN) in (A) patients without pneumonia (left first on the x-axis) and, in patients with pneumonia, before (second on the x-axis) and after (third on the x-axis) pneumonia onset; before the onset of pneumonia, there was no difference in PN compared to patients without pneumonia (OR = 1.01, 95% CI = 0.93–1.10), whereas after pneumonia onset, patients received more PN (OR = 1.19, 95% CI = 1.08–1.32); (B) mean amount of PN in patients without sepsis (left first on the x-axis) and, in patients with sepsis, before (second on the x-axis) and after (third on the x-axis) sepsis onset; before sepsis-onset, there was no difference in PN compared to patients without sepsis (OR = 1.98, 95% CI = 0.74–3.84), whereas after sepsis onset, patients received more PN (OR = 3.74, 95% CI = 2.65–9.48); statistical analysis was performed using a univariate linear model in generalized estimating equations with the amount of PN kilocalories as outcome variable. Error bars represent the standard error of mean. Kcal/kg/d = Kilocalories per kilogram bodyweight per day.

Supplemental PN was maintained for 8 (IQR 4–12) days, the mean amount was 12.5±0.6 kcal/kg/d. In Table 3, associations between the amount and duration of PN and hospital complications were assessed. Eight patients (20%) had no infectious complication, 24 (60%) had one and 8 patients (20%) had two infectious complications. Six patients (15%) developed sepsis. In three patients *staphylococcus species*, in one patient *Pseudomonas aeruginosa* and in one patient *Candida albicans* were cultured. In one patient, no pathogen could be identified. Catheter infection was detected in 13 patients (33%). Patients with catheter infection received PN for a longer period of time than patients without [16 (0–22) days vs. 3 (0–25) days, p<0.001]. Adjusted for length of ICU stay, this association was not significant anymore (p = 0.43). The amount of administered PN did not differ between patients with (13.2±0.9 kcal/kg/d) and without catheter infection (12.2±0.7 kcal/kg/d), p = 0.72. The relation between PN and severe infectious complications is further detailed in Fig 2. Patients with pneumonia or sepsis received more PN than patients without, however, after the onset of the infectious complication, but not before.

**Table 3:** Associations between supplemental parenteral nutrition and hospital complications.

	Duration of PN administration (days)			Amount of administered PN		
	Median (IQR) duration of PN administration (days)			Mean (SEM) of daily PN kcal/kg		
Complication	yes	no	p-value	yes	no	p-value
Delayed cerebral ischemia	8 (3–12)	8 (4–10)	0.63^#^	12.6 ± 1.0	12.5 ± 0.7	0.76^+^
Pneumonia	8 (5–12)	6 (3–11)	0.44^#^	13.4 ± 0.7	11.1 ± 0.9	0.16^+^
Sepsis	12 (5–13)	8 (3–10)	0.13^#^	19.5 ± 1.5	11.3 ± 0.6	0.05^+^
Ventriculitis	9.5 (n/a)	8 (4–11)	0.47^#^	11.6 ± 1.7	12.5 ± 0.6	0.91^+^
Urinary tract infection	10 (4–14)	8 (3–10)	0.24^#^	13.6 ± 1.1	12.2 ± 0.7	0.84^+^
One versus no infectious complication	8 (4–11)	4 (1–8)	0.11^#^	12.6 ± 0.7	7.6 ± 1.2	0.19^+^
Two versus no infectious complications	12 (5–14)	4 (1–8)	0.04^#^	16.7 ± 1.2	7.6 ± 1.2	0.05^+^
Renal Failure	6 (6–6)	8 (4–12)	0.72^#^	10.7 ± 1.8	12.6 ± 0.6	0.20^+^
Percutaneous dilatational tracheotomy	6 (4–12)	8 (4–11)	0.94^#^	12.0 ± 1.0	12.7 ± 1.7	0.80^+^
Length of mechanical ventilation	n/a	n/a	0.44^§^	n/a	n/a	0.42^&^
Length of ICU stay	n/a	n/a	0.24^§^	n/a	n/a	0.81^&^

Statistical analysis was performed using a univariate binary logistic (^#^) or linear (^§^) regression model with the respective complication as outcome variable for the assessment of associations between the duration of the administration of parenteral nutrition (PN) and hospital complications. Univariate binary logistic (^+^) or linear (^&^) modeling using generalized estimating equations with the respective complication as outcome variable was used to investigate associations between the daily amount of PN kilocalories (kcal) and complications. n/a = not applicable; ICU = intensive care unit.

### Protein

The mean daily protein provision was 1.17±0.02 g/kg. The amount of administered protein was significantly higher in patients who received supplemental PN (OR = 1.34, 95% CI = 1.17–1.55, p<0.001) and on days with PN (OR = 1.6, 95% CI = 1.48–1.72, p<0.001). Patients, in whom nutrition goal 1 or nutrition goal 2 was accomplished, received significantly less protein compared to patients in whom the respective goal was not reached (mean over study period 1.02±0.02 versus 1.29±0.03 g/kg/d; GEE, OR = 0.8, 95% CI = 0.69–0.92 p = 0.002; and 1.021±0.025 versus 1.27±0.03 g/kg/d; GEE, OR = 0.76, 95% CI = 0.66–0.87, p<0.001). Analyses were adjusted for Hunt and Hess grade, age, modified Fisher score and gender.

The mean protein administration by intravenous amino acid supplementation on days 1–3 was 0.39±0.04 g/kg, 0.28±0.05 g/kg and 0.19±0.05 g/kg, 56%, 30% and 19% of the total daily protein administration, respectively.

### Nutrition and functional outcome

Three months after SAH, 12 patients (30%) had a favorable (mRS 0–2) and 28 patients (70%) poor functional outcome (mRS 3–6). Neither the amount of EN calories (p = 0.21), nor total calories (p = 0.64) was associated with functional outcome.

**Fig 3:**
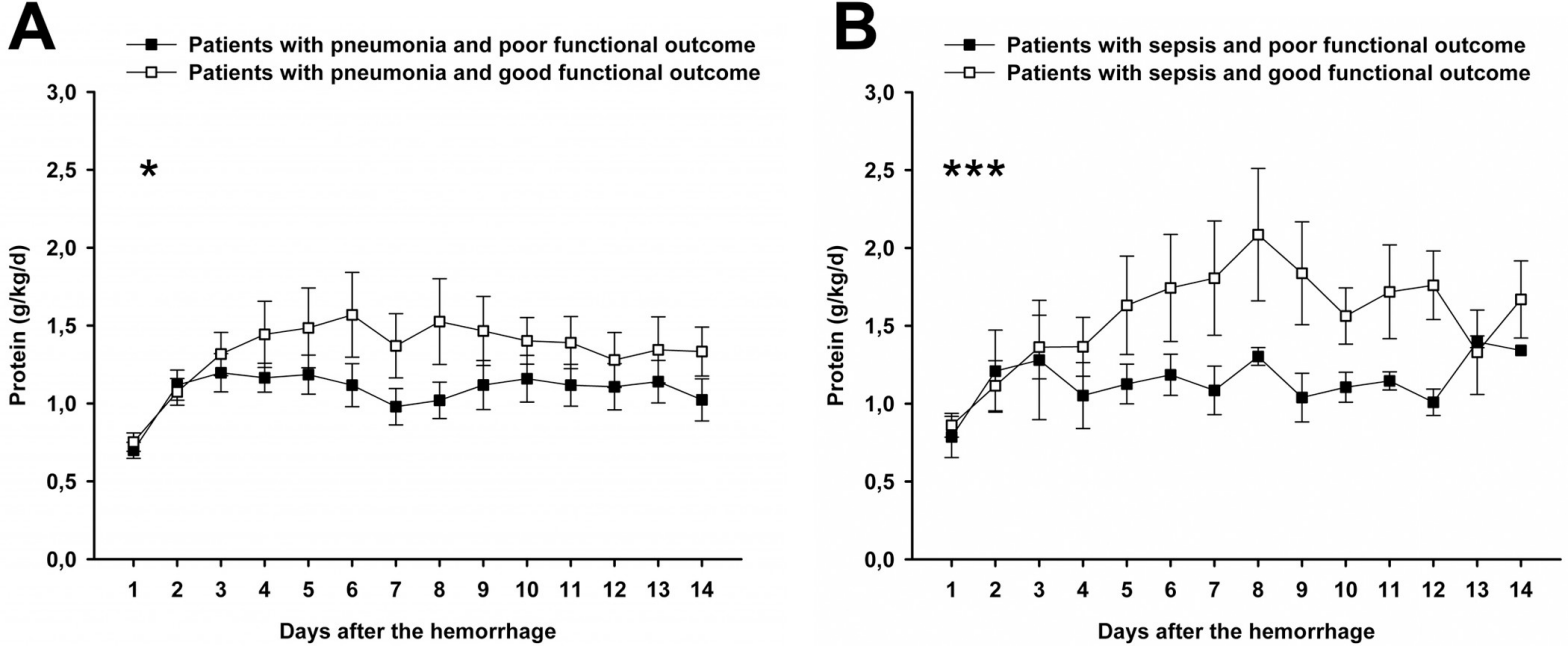
Protein administration and functional outcome in patients with severe infectious complications. In patients with pneumonia (A; adj. OR = 1.084 for good outcome per g/kg/d, 95% CI = 1.007–1.17, p = 0.031) or sepsis (B; adj. OR = 1.2 for good outcome per g/kg/d, 95% CI = 1.18–1.23, p<0.001) there was a significant association between higher protein administration and good functional outcome (modified Rankin Scale score ≤2). Circles show the mean grams per kilogram bodyweight per day (g/kg/d) of protein. Error bars indicate the standard error of mean. Asterisks indicate the level of significance (*p<0.05, ***p<0.001) of the binary logistic model in a generalized estimating equation with functional outcome as dichotomized outcome variable. The analyses were adjusted for age, Hunt & Hess grade, gender and modified Fisher grade.

Patients with good functional outcome received more supplemental PN than patients with poor outcome (mean over study period 15.1±1.1 versus 11.3±0.6 kcal/kg/d; GEE adj. OR = 1.02 for good outcome per kcal/kg/d, 95% CI = 1.004–1.03, p = 0.046), adjusted for Hunt and Hess grade, age, modified Fisher score and gender. In the overall cohort, higher protein administration was not significantly associated with good outcome (mean over study period 1.29±0.04 versus 1.11±0.02 g/kg/d; GEE, OR = 1.07, 95% CI = 0.992–1.15, p = 0.08). In the subgroups of patients with pneumonia (adj. OR = 1.084 for good outcome per g/kg/d, 95% CI = 1.007–1.17, p = 0.031) or sepsis (adj. OR = 1.2 for good outcome per g/kg/d, 95% CI = 1.18–1.23, p<0.001), there was an association between higher protein intake and good functional outcome (Fig 3).

## Discussion

We found that enteral feeding was not sufficient to provide adequate calories and protein in SAH patients. Supplemental PN led to a reliable accomplishment of nutrition goals and was associated with increased protein administration. Patients with good functional outcome received more supplemental PN compared to patients with poor outcome. A higher protein supply was associated with good functional outcome in patients with sepsis or pneumonia.

The inability of EN to provide calculated caloric and protein requirements in SAH patients has been reported earlier [9, 24, 37]. Our data indicate that mainly secondary (including infectious) complications were associated with a failure in reaching nutritional goals using EN. Some studies interpreted insufficient EN as a risk factor for hospital infections [9, 24]. However, infectious complications and concomitant inflammatory responses may also contribute to gastro-intestinal dysmotility [38]. In this cohort, there was no difference in calories that could be applied by EN between patients with early and late onset pneumonia. As early onset pneumonia is predominantly caused by aspiration, it is conceivable that pneumonia is rather the reason for, but not the consequence of, impaired enteral feeding. Our observation that supplemental PN had to be increased only after the diagnosis of pneumonia or sepsis was established, but the amount did not differ before diagnosis, further supports the assumption, that infectious complications precede the impairment of enteral feeding.

Acute hydrocephalus is common in SAH patients and is associated with raised intracranial pressure (ICP) in the acute phase. Elevated ICP was associated with reduced enteral feeding tolerance [39], which may explain the lower amount of EN in patients with hydrocephalus. Continuous sedation and analgesia is a common strategy in neuro-critical care patients to prevent or treat raised ICP [40]. The negative effects of opioids on intestinal motility are well described [41]. High doses of both, midazolam and sufentanil, were associated with decreased EN tolerance in our patients, as were higher doses of vasopressors. Supplemental PN may improve the provision of calories and protein in SAH patients requiring deep anesthesia and vasopressors.

There was no association between PN and DCI, medical complications or length of mechanical ventilation and ICU stay. As patients with infectious complications received more PN for a longer period of time, we further investigated the association of PN with pneumonia and sepsis. Until the diagnosis of pneumonia or sepsis was made, there was no difference in the amount of PN received by patients who would later develop these complications and those who would not. Therefore, a causal relation between PN administration and severe infectious complications seems unlikely. Enteral nutrition is involved in preventing intestinal mucosal permeability and bacterial translocation [42, 43]. Importantly, we did not detect intestinal bacteria as etiologic pathogens in patients developing sepsis. Catheter infection was common in this cohort and it was associated with the length of PN application. It is worth noting, however, that after the adjustment for length of ICU stay, this association was not significant. Therefore, catheter infection may reflect the longer need for a central line in general.

Whether or not and in which patient population a caloric deficit can be tolerated has been in the focus of recent research. Permissive underfeeding (but providing adequate amounts of protein) was not associated with worse outcome in predominantly medical and surgical patients [15]. In TBI patients, however, there is evidence that an increased early provision of calculated requirements is associated with reduced mortality [4]. In SAH patients, no association between energy balance and functional outcome was found [9]. In line with this, there was no association between the total amount of calories provided and functional outcome in our population.

Contrarily, we found an association between higher doses of PN and good functional outcome. The utility and best time point of initiation of supplemental PN in critically ill patients remains uncertain. The EPaNIC trial detected a prolonged ICU length of stay and an increased rate of complications in patients receiving early (during the first 7 days) supplemental PN [12]. Some factors may, however, limit the applicability of these findings to our cohort. Poor-grade SAH patients requiring enteral/parenteral nutrition, as included in our study, typically have a much longer ICU length of stay than patients included in the EPaNIC trial (33 versus 3–4 days). Furthermore, we did not apply a tight glycemic control protocol, as this was associated with impaired brain metabolism in patients with acute brain injury [44]. Moreover, our patients showed less organ dysfunction and chronic morbidity on admission, indicated by a lower APACHE II score.

By using supplemental PN, we administered a considerably higher dose of protein than previously reported in SAH patients [23, 37]. Higher protein intake was associated with decreased mortality [16–18], more ventilator-free days [19], an improvement in sequential organ failure assessment (SOFA) score [20], and preserved muscle strength [21] in mixed populations. We found a trend towards better functional outcome with higher protein administration in our cohort, which did not reach statistical significance. In patients with sepsis or pneumonia, however, higher daily protein supply was significantly associated with good functional outcome. This may be due to the important role of protein in maintaining immune functions [22], and contribute to the association between more PN and good outcome in our patients. In an earlier study on sepsis patients (ICU admission diagnosis), however, no benefit of achieving protein goals was found [18].

Whether a higher protein dose via the enteral route could improve outcome in SAH patients remains elusive and should be investigated in a prospective interventional trial. Our findings that patients with sepsis or pneumonia, the subgroups in which we found an association between high-protein feeding and good outcome, showed significantly decreased EN tolerance should be taken into account. Furthermore, protein digestion and absorption in the gut is energy dependent [45]. A sympathetic stress response occurs secondary to SAH, during which elevated adrenalin levels may lead to reduced gastrointestinal perfusion [46, 47], and therefore a lack of energy. Thus, the uptake of EN protein may be incomplete. We found borderline significant associations between a longer time of administration and a larger amount of PN and more infectious complications. However, this association was only present after the complications occurred, which illustrates that supplemental PN was reactively increased when EN was not well tolerated. Therefore, supplemental PN seems to be an effective and safe method for increasing protein delivery in SAH patients.

Some limitations of our study merit consideration. Most importantly, the design of this study was observational and data analysis was retrospective, thus we cannot conclude causality. Importantly however, following our nutrition protocol, supplemental PN was only given or increased when EN was considered insufficient. We also described that hospital complications were associated with less EN tolerance, necessitating larger amounts of supplemental PN. Therefore the association between more PN and good outcome is not due to patients with favorable clinical course accidently receiving more parenteral supplementation. Furthermore our final sample size was rather small. It is important to note that, by excluding patients who did not receive artificial nutrition, a selection towards poor-grade SAH patients occurred (60% Hunt and Hess grade 4 or 5), which is also represented by the high rate of infectious complications and the ICU length of stay. We did not perform indirect calorimetry or urine nitrogen measurements, thus we cannot provide data on energy and nitrogen balance. Furthermore, we did not quantify calories and protein from oral food intake, therefore data had to be excluded as soon as patients were allowed to eat.

## Conclusions

Enteral nutrition was insufficient to deliver the calculated amount of energy and protein requirements in subarachnoid hemorrhage patients. Supplemental PN was safe and associated with a reliable accomplishment of nutrition goals as well as higher protein administration. Patients with good functional outcome received more supplemental PN compared to patients with poor outcome. A higher protein supply was associated with good functional outcome in patients with sepsis or pneumonia.

## Supporting information

S1 Fig **S1 Fig describes the patient exclusion process.** SAH = subarachnoid hemorrhage; ICU = intensive care unit; EN = enteral nutrition; H&H = Hunt & Hess grade.
(PDF)

## References

[1] Singer P, Blaser AR, Berger MM, Alhazzani W, Calder PC, Casaer MP, et al. ESPEN guideline on clinical nutrition in the intensive care unit. Clin Nutr. 2019;38(1):48–79. Epub 2018/10/24. doi: 10.1016/j.clnu.2018.08.037 . 30348463

[2] McClave SA, Taylor BE, Martindale RG, Warren MM, Johnson DR, Braunschweig C, et al. Guidelines for the Provision and Assessment of Nutrition Support Therapy in the Adult Critically Ill Patient: Society of Critical Care Medicine (SCCM) and American Society for Parenteral and Enteral Nutrition (A.S.P.E.N.). JPEN J Parenter Enteral Nutr. 2016;40(2):159–211. Epub 2016/01/17. doi: 10.1177/0148607115621863 . 26773077

[3] Zusman O, Theilla M, Cohen J, Kagan I, Bendavid I, Singer P. Resting energy expenditure, calorie and protein consumption in critically ill patients: a retrospective cohort study. Crit Care. 2016;20(1):367. Epub 2016/11/12. doi: 10.1186/s13054-016-1538-4 ; PubMed Central PMCID: PMC5105237. 27832823 PMC5105237

[4] Hartl R, Gerber LM, Ni Q, Ghajar J. Effect of early nutrition on deaths due to severe traumatic brain injury. J Neurosurg. 2008;109(1):50–6. Epub 2008/07/02. doi: 10.3171/JNS/2008/109/7/0050 . 18590432

[5] Harvey SE, Parrott F, Harrison DA, Bear DE, Segaran E, Beale R, et al. Trial of the route of early nutritional support in critically ill adults. N Engl J Med. 2014;371(18):1673–84. Epub 2014/10/02. doi: 10.1056/NEJMoa1409860 . 25271389

[6] Reignier J, Boisrame-Helms J, Brisard L, Lascarrou JB, Ait Hssain A, Anguel N, et al. Enteral versus parenteral early nutrition in ventilated adults with shock: a randomised, controlled, multicentre, open-label, parallel-group study (NUTRIREA-2). Lancet. 2018;391(10116):133–43. Epub 2017/11/13. doi: 10.1016/S0140-6736(17)32146-3 . 29128300

[7] Arabi YM, Casaer MP, Chapman M, Heyland DK, Ichai C, Marik PE, et al. The intensive care medicine research agenda in nutrition and metabolism. Intensive Care Med. 2017;43(9):1239–56. Epub 2017/04/05. doi: 10.1007/s00134-017-4711-6 ; PubMed Central PMCID: PMC5569654. 28374096 PMC5569654

[8] Heyland DK, Schroter-Noppe D, Drover JW, Jain M, Keefe L, Dhaliwal R, et al. Nutrition support in the critical care setting: current practice in canadian ICUs—opportunities for improvement? JPEN J Parenter Enteral Nutr. 2003;27(1):74–83. Epub 2003/01/29. doi: 10.1177/014860710302700174 . 12549603

[9] Badjatia N, Fernandez L, Schlossberg MJ, Schmidt JM, Claassen J, Lee K, et al. Relationship between energy balance and complications after subarachnoid hemorrhage. JPEN J Parenter Enteral Nutr. 2010;34(1):64–9. Epub 2009/11/04. doi: 10.1177/0148607109348797 . 19884354

[10] Kutsogiannis J, Alberda C, Gramlich L, Cahill NE, Wang M, Day AG, et al. Early use of supplemental parenteral nutrition in critically ill patients: results of an international multicenter observational study. Crit Care Med. 2011;39(12):2691–9. Epub 2011/07/19. doi: 10.1097/CCM.0b013e3182282a83 . 21765355

[11] Heidegger CP, Berger MM, Graf S, Zingg W, Darmon P, Costanza MC, et al. Optimisation of energy provision with supplemental parenteral nutrition in critically ill patients: a randomised controlled clinical trial. Lancet. 2013;381(9864):385–93. Epub 2012/12/12. doi: 10.1016/S0140-6736(12)61351-8 . 23218813

[12] Casaer MP, Mesotten D, Hermans G, Wouters PJ, Schetz M, Meyfroidt G, et al. Early versus late parenteral nutrition in critically ill adults. N Engl J Med. 2011;365(6):506–17. Epub 2011/07/01. doi: 10.1056/NEJMoa1102662 . 21714640

[13] Heyland DK. Early supplemental parenteral nutrition in critically ill adults increased infections, ICU length of stay and cost. Evid Based Med. 2012;17(3):86–7. Epub 2011/10/27. doi: 10.1136/ebm.2011.100252 . 22028368

[14] Alsharif DJ, Alsharif FJ, Aljuraiban GS, Abulmeaty MMA. Effect of Supplemental Parenteral Nutrition Versus Enteral Nutrition Alone on Clinical Outcomes in Critically Ill Adult Patients: A Systematic Review and Meta-Analysis of Randomized Controlled Trials. Nutrients. 2020;12(10). Epub 2020/10/02. doi: 10.3390/nu12102968 ; PubMed Central PMCID: PMC7601814. 32998412 PMC7601814

[15] Arabi YM, Aldawood AS, Haddad SH, Al-Dorzi HM, Tamim HM, Jones G, et al. Permissive Underfeeding or Standard Enteral Feeding in Critically Ill Adults. N Engl J Med. 2015;372(25):2398–408. Epub 2015/05/21. doi: 10.1056/NEJMoa1502826 . 25992505

[16] Nicolo M, Heyland DK, Chittams J, Sammarco T, Compher C. Clinical Outcomes Related to Protein Delivery in a Critically Ill Population: A Multicenter, Multinational Observation Study. JPEN J Parenter Enteral Nutr. 2016;40(1):45–51. Epub 2015/04/23. doi: 10.1177/0148607115583675 . 25900319

[17] Weijs PJ, Stapel SN, de Groot SD, Driessen RH, de Jong E, Girbes AR, et al. Optimal protein and energy nutrition decreases mortality in mechanically ventilated, critically ill patients: a prospective observational cohort study. JPEN J Parenter Enteral Nutr. 2012;36(1):60–8. Epub 2011/12/15. doi: 10.1177/0148607111415109 . 22167076

[18] Weijs PJ, Looijaard WG, Beishuizen A, Girbes AR, Oudemans-van Straaten HM. Early high protein intake is associated with low mortality and energy overfeeding with high mortality in non-septic mechanically ventilated critically ill patients. Crit Care. 2014;18(6):701. Epub 2014/12/17. doi: 10.1186/s13054-014-0701-z ; PubMed Central PMCID: PMC4279460. 25499096 PMC4279460

[19] Elke G, Wang M, Weiler N, Day AG, Heyland DK. Close to recommended caloric and protein intake by enteral nutrition is associated with better clinical outcome of critically ill septic patients: secondary analysis of a large international nutrition database. Crit Care. 2014;18(1):R29. Epub 2014/02/11. doi: 10.1186/cc13720 ; PubMed Central PMCID: PMC4056527. 24506888 PMC4056527

[20] Rugeles SJ, Rueda JD, Diaz CE, Rosselli D. Hyperproteic hypocaloric enteral nutrition in the critically ill patient: A randomized controlled clinical trial. Indian J Crit Care Med. 2013;17(6):343–9. Epub 2014/02/07. doi: 10.4103/0972-5229.123438 ; PubMed Central PMCID: PMC3902568. 24501485 PMC3902568

[21] Ferrie S, Allman-Farinelli M, Daley M, Smith K. Protein Requirements in the Critically Ill: A Randomized Controlled Trial Using Parenteral Nutrition. JPEN J Parenter Enteral Nutr. 2016;40(6):795–805. Epub 2015/12/05. doi: 10.1177/0148607115618449 . 26635305

[22] Weijs PJ, Cynober L, DeLegge M, Kreymann G, Wernerman J, Wolfe RR. Proteins and amino acids are fundamental to optimal nutrition support in critically ill patients. Crit Care. 2014;18(6):591. Epub 2015/01/08. doi: 10.1186/s13054-014-0591-0 ; PubMed Central PMCID: PMC4520087. 25565377 PMC4520087

[23] Badjatia N, Monahan A, Carpenter A, Zimmerman J, Schmidt JM, Claassen J, et al. Inflammation, negative nitrogen balance, and outcome after aneurysmal subarachnoid hemorrhage. Neurology. 2015;84(7):680–7. Epub 2015/01/18. doi: 10.1212/WNL.0000000000001259 ; PubMed Central PMCID: PMC4336106. 25596503 PMC4336106

[24] Cinotti R, Dordonnat-Moynard A, Feuillet F, Roquilly A, Rondeau N, Lepelletier D, et al. Risk factors and pathogens involved in early ventilator-acquired pneumonia in patients with severe subarachnoid hemorrhage. Eur J Clin Microbiol Infect Dis. 2014;33(5):823–30. Epub 2013/12/11. doi: 10.1007/s10096-013-2020-8 . 24322991

[25] Taylor SJ, Fettes SB, Jewkes C, Nelson RJ. Prospective, randomized, controlled trial to determine the effect of early enhanced enteral nutrition on clinical outcome in mechanically ventilated patients suffering head injury. Crit Care Med. 1999;27(11):2525–31. Epub 1999/12/01. doi: 10.1097/00003246-199911000-00033 . 10579275

[26] Perel P, Yanagawa T, Bunn F, Roberts I, Wentz R, Pierro A. Nutritional support for head-injured patients. Cochrane Database Syst Rev. 2006;(4):CD001530. Epub 2006/10/21. doi: 10.1002/14651858.CD001530.pub2 ; PubMed Central PMCID: PMC7025778. 17054137 PMC7025778

[27] Hunt WE, Hess RM. Surgical risk as related to time of intervention in the repair of intracranial aneurysms. J Neurosurg. 1968;28(1):14–20. Epub 1968/01/01. doi: 10.3171/jns.1968.28.1.0014 . 5635959

[28] Knaus WA, Draper EA, Wagner DP, Zimmerman JE. APACHE II: a severity of disease classification system. Crit Care Med. 1985;13(10):818–29. Epub 1985/10/01. . 3928249

[29] Claassen J, Bernardini GL, Kreiter K, Bates J, Du YE, Copeland D, et al. Effect of cisternal and ventricular blood on risk of delayed cerebral ischemia after subarachnoid hemorrhage: the Fisher scale revisited. Stroke. 2001;32(9):2012–20. Epub 2001/09/08. doi: 10.1161/hs0901.095677 . 11546890

[30] Steiner T, Juvela S, Unterberg A, Jung C, Forsting M, Rinkel G, et al. European Stroke Organization guidelines for the management of intracranial aneurysms and subarachnoid haemorrhage. Cerebrovasc Dis. 2013;35(2):93–112. Epub 2013/02/15. doi: 10.1159/000346087 . 23406828

[31] Connolly ES Jr., Rabinstein AA, Carhuapoma JR, Derdeyn CP, Dion J, Higashida RT, et al. Guidelines for the management of aneurysmal subarachnoid hemorrhage: a guideline for healthcare professionals from the American Heart Association/american Stroke Association. Stroke. 2012;43(6):1711–37. Epub 2012/05/05. doi: 10.1161/STR.0b013e3182587839 . 22556195

[32] Bederson JB, Connolly ES Jr., Batjer HH, Dacey RG, Dion JE, Diringer MN, et al. Guidelines for the management of aneurysmal subarachnoid hemorrhage: a statement for healthcare professionals from a special writing group of the Stroke Council, American Heart Association. Stroke. 2009;40(3):994–1025. Epub 2009/01/24. doi: 10.1161/STROKEAHA.108.191395 . 19164800

[33] Vergouwen MD, Vermeulen M, van Gijn J, Rinkel GJ, Wijdicks EF, Muizelaar JP, et al. Definition of delayed cerebral ischemia after aneurysmal subarachnoid hemorrhage as an outcome event in clinical trials and observational studies: proposal of a multidisciplinary research group. Stroke. 2010;41(10):2391–5. Epub 2010/08/28. doi: 10.1161/STROKEAHA.110.589275 . 20798370

[34] Horan TC, Andrus M, Dudeck MA. CDC/NHSN surveillance definition of health care-associated infection and criteria for specific types of infections in the acute care setting. Am J Infect Control. 2008;36(5):309–32. Epub 2008/06/10. doi: 10.1016/j.ajic.2008.03.002 . 18538699

[35] Dellinger RP, Levy MM, Rhodes A, Annane D, Gerlach H, Opal SM, et al. Surviving sepsis campaign: international guidelines for management of severe sepsis and septic shock: 2012. Crit Care Med. 2013;41(2):580–637. Epub 2013/01/29. doi: 10.1097/CCM.0b013e31827e83af . 23353941

[36] American Thoracic S, Infectious Diseases Society of A. Guidelines for the management of adults with hospital-acquired, ventilator-associated, and healthcare-associated pneumonia. Am J Respir Crit Care Med. 2005;171(4):388–416. Epub 2005/02/09. doi: 10.1164/rccm.200405-644ST . 15699079

[37] Schmidt JM, Claassen J, Ko SB, Lantigua H, Presciutti M, Lee K, et al. Nutritional support and brain tissue glucose metabolism in poor-grade SAH: a retrospective observational study. Crit Care. 2012;16(1):R15. Epub 2012/01/27. doi: 10.1186/cc11160 ; PubMed Central PMCID: PMC3396251. 22277085 PMC3396251

[38] Ukleja A. Altered GI motility in critically Ill patients: current understanding of pathophysiology, clinical impact, and diagnostic approach. Nutr Clin Pract. 2010;25(1):16–25. Epub 2010/02/05. doi: 10.1177/0884533609357568 . 20130154

[39] Norton JA, Ott LG, McClain C, Adams L, Dempsey RJ, Haack D, et al. Intolerance to enteral feeding in the brain-injured patient. J Neurosurg. 1988;68(1):62–6. Epub 1988/01/01. doi: 10.3171/jns.1988.68.1.0062 . 3121807

[40] Helbok R, Kurtz P, Schmidt MJ, Stuart MR, Fernandez L, Connolly SE, et al. Effects of the neurological wake-up test on clinical examination, intracranial pressure, brain metabolism and brain tissue oxygenation in severely brain-injured patients. Crit Care. 2012;16(6):R226. Epub 2012/11/29. doi: 10.1186/cc11880 ; PubMed Central PMCID: PMC3672610. 23186037 PMC3672610

[41] Holzer P. New approaches to the treatment of opioid-induced constipation. Eur Rev Med Pharmacol Sci. 2008;12 Suppl 1:119–27. Epub 2008/10/18. ; PubMed Central PMCID: PMC4370832. 18924451 PMC4370832

[42] Kang W, Kudsk KA. Is there evidence that the gut contributes to mucosal immunity in humans? JPEN J Parenter Enteral Nutr. 2007;31(3):246–58. Epub 2007/04/28. doi: 10.1177/0148607107031003246 . 17463152

[43] Marik PE, Zaloga GP. Early enteral nutrition in acutely ill patients: a systematic review. Crit Care Med. 2001;29(12):2264–70. Epub 2002/01/22. doi: 10.1097/00003246-200112000-00005 . 11801821

[44] Oddo M, Schmidt JM, Carrera E, Badjatia N, Connolly ES, Presciutti M, et al. Impact of tight glycemic control on cerebral glucose metabolism after severe brain injury: a microdialysis study. Crit Care Med. 2008;36(12):3233–8. Epub 2008/10/22. doi: 10.1097/CCM.0b013e31818f4026 . 18936695

[45] Silk DB. Digestion and absorption of carbohydrate protein and fat. Contemp Issues Clin Biochem. 1986;4:7–40. Epub 1986/01/01. 3107903

[46] Meier-Hellmann A, Reinhart K. Effects of catecholamines on regional perfusion and oxygenation in critically ill patients. Acta Anaesthesiol Scand Suppl. 1995;107:239–48. Epub 1995/01/01. doi: 10.1111/j.1399-6576.1995.tb04365.x 8599285

[47] Naredi S, Lambert G, Eden E, Zall S, Runnerstam M, Rydenhag B, et al. Increased sympathetic nervous activity in patients with nontraumatic subarachnoid hemorrhage. Stroke. 2000;31(4):901–6. Epub 2001/02/07. doi: 10.1161/01.str.31.4.901 . 10753996

