# Supplementary material for: Early supplemental parenteral nutrition for the achievement of nutritional goals in subarachnoid hemorrhage patients: An observational cohort study
Source: PLoS One. 2022 Mar 18;17(3):e0265729. doi: 10.1371/journal.pone.0265729 (PMC8932621; doi:10.1371/journal.pone.0265729)
Supplement: S1 Fig — describes the patient exclusion process. SAH = subarachnoid hemorrhage; ICU = intensive care unit; EN = enteral nutrition; H&H = Hunt & Hess grade. (PDF) [file pone.0265729.s001.pdf]

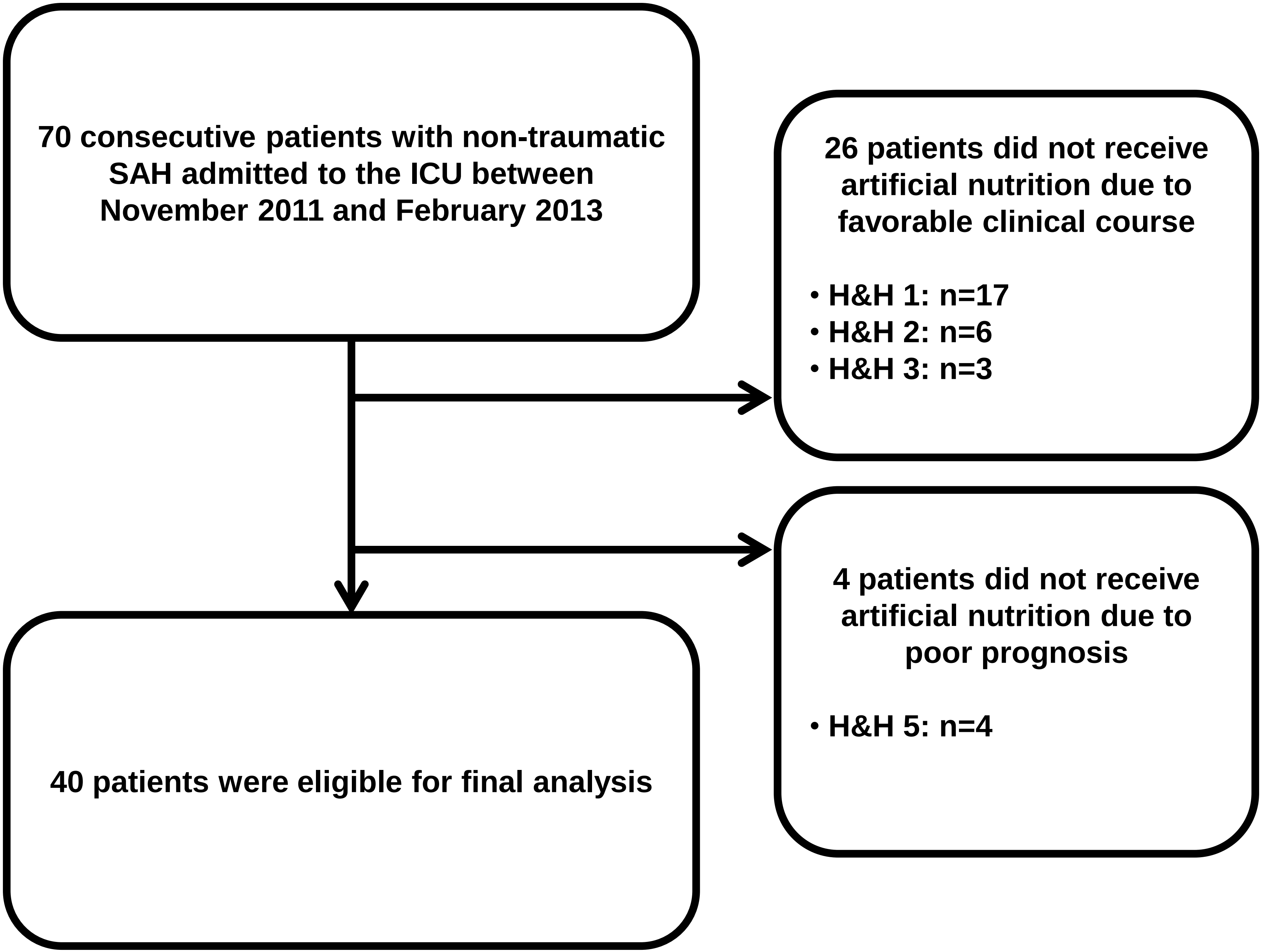
70 consecutive patients with non-traumatic SAH admitted to the ICU between November 2011 and February 2013
26 patients did not receive artificial nutrition due to favorable clinical course
• H&H 1: n=17
• H&H 2: n=6
• H&H 3: n=3
4 patients did not receive artificial nutrition due to poor prognosis
• H&H 5: n=4
40 patients were eligible for final analysis
